# Supplementary material for: Content-rich biological network constructed by mining PubMed abstracts
Source: BMC Bioinformatics. 2004 Oct 8;5:147. doi: 10.1186/1471-2105-5-147 (PMC528731; doi:10.1186/1471-2105-5-147)
Supplement: Additional File 2 — The original results of the above study (non-essential files are deleted to keep the file size under the limit set by BMC bioinformatics). [file 1471-2105-5-147-S2.bz2 › chilibotAdditionalFile2/dip20/483ID8702798E10574/html/IL8RB_PPBP.html]

 


 **IL8RB** and **PPBP** 
  
Found 28 abstracts in PubMed, retrieved 20.  
 

 What does Google say? 
 PDF only 
| .edu only 

---

**Interactive relationship** (e.g. stimulation, inhibition, etc)

**Neutral relationship**- Although two IL 8R types are expressed by PMN, only CXCR2  [ **IL8RB** ]  binds NAP 2  [ **PPBP** ]  and IL 8 with equally high affinity.  Ref: 9190946 J Immunol, 1997
- The migration activating concentrations of 50 ng mL GCP 2 and NAP 2  [ **PPBP** ]  induced less potent migratory responses and FAK phosphorylation in CXCR2  [ **IL8RB** ]  expressing cells as compared with IL 8.  Ref: 12627953 Biochemistry, 2003
- All six agonists for  **IL8RB**  competed for high affinity 125I IL 8, GROalpha, NAP 2  [ **PPBP** ] , and ENA 78 binding sites at  **IL8RB** .  Ref: 8702798 J Biol Chem, 1996
- In conclusion, high affinity binding of IL 8, NAP 2  [ **PPBP** ] , and GROalpha to CXCR2  [ **IL8RB** ]  involves interaction with specific and different amino acid residues of CXCR2  [ **IL8RB** ] .  Ref: 11023662 Cytokine, 2000
- Immunoprecipitation and Western blot analyses of surface expressed receptors covalently linked to IL 8 or NAP 2  [ **PPBP** ]  as well as in their unloaded state revealed the occurrence of a single CXCR 2  [ **IL8RB** ]  variant with an apparent size of 56 kDa.  Ref: 10878382 J Immunol, 2000
- Moreover, although CXCR2  [ **IL8RB** ]  bound IL 8 and NAP 2  [ **PPBP** ]  with similarly high affinity, IL 8 functionally competed with and displaced NAP 2  [ **PPBP** ] , and prompted high levels of internalization, similar to those induced by IL 8 alone.  Ref: 10623425 Cytokine, 1999
- Similar to other members of this group, such as IL 8, NAP 2  [ **PPBP** ]  activates chemotaxis and degranulation in neutrophils polymorphonuclear PMN through chemokine receptors CXCR 1 and CXCR 2  [ **IL8RB** ] .  Ref: 11049972 Blood, 2000
- The rank order of agonist potency, based on inspection of the mean effective concentration values EC50, for  **IL8RB**  was GROgamma 1 nM > IL 8 4 nM approximately GROalpha 5 nM approximately GRObeta 4 nM approximately NAP 2  [ **PPBP** ]  7 nM > ENA 78 11 nM, and for IL8RA  [ **IL8RB** ]  was IL 8 4 nM >>> ENA 78 40 nM approximately NAP 2  [ **PPBP** ]  45 nM > GROalpha 63 nM approximately GROgamma 65 nM >> GRObeta.  Ref: 8702798 J Biol Chem, 1996
- CXCR1 and CXCR2  [ **IL8RB** ]  mediate migratory activities in response to IL 8 and other ELR CXC chemokines e.g., GCP 2 and NAP 2  [ **PPBP** ] .  Ref: 12627953 Biochemistry, 2003
- One such receptor, CXCR2  [ **IL8RB** ] , binds multiple CXC chemokines, including interleukin 8, GRO alpha, GRO beta, GRO gamma, and NAP 2  [ **PPBP** ] .  Ref: 10747307 Arch Pathol Lab Med, 2000
- The G protein coupled CXC chemokine receptor CXCR 2  [ **IL8RB** ]  mediates activation of neutrophil effector functions in response to multiple ligands, including IL 8 and neutrophil activating peptide 2 NAP 2  [ **PPBP** ] .  Ref: 10878382 J Immunol, 2000
- These results provide evidence that both receptors are involved in NAP 2  [ **PPBP** ]  induced neutrophil chemotaxis, with CXCR 2  [ **IL8RB** ]  rendering the cells responsive to low dosages of the chemokine, and with CXCR 1 extending their responsiveness to NAP 2  [ **PPBP** ]  dosages higher by several orders of magnitude.  Ref: 9373270 Blood, 1997
- CXCR2  [ **IL8RB** ]  binds IL 8 and other structurally related chemokines such as growth related oncogene GRO a, GRObeta, GROgamma, neutrophil activating peptide 2 NAP 2  [ **PPBP** ]  and epithelial cell derived neutrophil activating peptide 78 ENA 78.  Ref: 12365735 Fish Shellfish Immunol, 2002
- Phosphorylation analysis shows that IL 8 induces higher levels of phosphorylation of the carboxyl terminus of CXCR2  [ **IL8RB** ]  than does NAP 2  [ **PPBP** ] , suggesting that the level of phosphorylation contributes to the ability of the chemokines to attenuate the chemotactic response.  Ref: 9190946 J Immunol, 1997
- After phagocytosis of opsonized yeast, the expression of CXCR1 and CXCR2  [ **IL8RB** ]  was substantially down regulated and was accompanied by reduced Ca responses to corresponding ligands, IL 8 and neutrophil activating peptide 2 NAP 2  [ **PPBP** ] .  Ref: 12239185 Blood, 2002
- These receptors did not participate in ligand induced receptor trafficking, while surface expressed CXCR 2  [ **IL8RB** ]  was internalized and re expressed following stimulation with NAP 2  [ **PPBP** ] .  Ref: 10878382 J Immunol, 2000

**Non-interactive relationship** (e.g. studied together, co-existance, homology, etc.)

- Investigating the involvement of the two chemokine receptors CXCR 1 and CXCR 2  [ **IL8RB** ]  in NAP 2  [ **PPBP** ]  mediated chemotaxis, we observe that the cells become desensitized to the first optimum of the chemokine after selective downregulation of CXCR 2  [ **IL8RB** ] , while both optima disappear upon simultaneous downregulation of both receptors.  Ref: 9373270 Blood, 1997
- By enzymatic removal of one 9 kDa carbohydrate moiety in surface expressed CXCR 2  [ **IL8RB** ]  we can show that neither NAP 2  [ **PPBP** ]  induced trafficking nor signaling of the receptor is dependent on its full glycosylation.  Ref: 10878382 J Immunol, 2000
- The objective of this investigation was to determine the amino acid residues of the human neutrophil CXC chemokine receptor 2 CXCR2  [ **IL8RB** ]  that are critical for binding the ligands interleukin 8 IL 8, neutrophil activating peptide 2 NAP 2  [ **PPBP** ] , and growth related protein alpha GROalpha and critical for receptor mediated signal transduction.  Ref: 11023662 Cytokine, 2000
- Correspondingly, we found PBP  [ **PPBP** ]  and, less potently, CTAP III to inhibit CXCR 2  [ **IL8RB** ]  but not CXCR 1 dependent chemotaxis of neutrophils toward NAP 2  [ **PPBP** ] .  Ref: 11049972 Blood, 2000
- Using HEK 293 cell transfectants, IL 8 was shown to induce significantly higher levels of CXCR2  [ **IL8RB** ]  internalization than NAP 2  [ **PPBP** ] .  Ref: 10623425 Cytokine, 1999
- Consistent with this conclusion is inhibition of the chemotactic response to monocytes by agents that block CXCR2  [ **IL8RB** ] , the receptor that NAP 2  [ **PPBP** ]  uses.  Ref: 12101278 J Leukoc Biol, 2002
- By using enriched CXCR2  [ **IL8RB** ]  transfected 293 cells, we show that high doses of IL 8 induce attenuation of chemotaxis, while equivalent doses of NAP 2  [ **PPBP** ]  do not.  Ref: 9190946 J Immunol, 1997
- Blocking monoclonal antibodies MoAbs specific for CXCR 2  [ **IL8RB** ]  or CXCR 1 either suppress the first optimum of NAP 2  [ **PPBP** ]  induced chemotaxis or drastically reduce the second one, respectively.  Ref: 9373270 Blood, 1997
- The differential control of CXCR1 vs CXCR2  [ **IL8RB** ]  internalization by IL 8 and NAP 2  [ **PPBP** ] , as well as by phosphorylation mediated mechanisms, suggests that a chemokine and receptor specific mode of regulation of internalization may contribute to the divergent activities of these receptors.  Ref: 10623425 Cytokine, 1999
- Transendothelial migration of neutrophils in response to CXCL7  [ **PPBP** ]  or IL 8 CXCL8 was subject to modulation by CTAP III, but not CXCL4, as seen by drastic desensitization of the migratory response of neutrophils pre exposed to CTAP III, which was paralleled by selective down modulation of CXCR 2  [ **IL8RB** ] .  Ref: 12193731 J Immunol, 2002
- GROalpha, GRObeta, GROgamma, NAP 2  [ **PPBP** ] , and ENA 78 competed weakly for the high affinity IL 8 binding site at IL8RA  [ **IL8RB** ] .  Ref: 8702798 J Biol Chem, 1996
- This study was undertaken to define the regions of the human interleukin 8 type B receptor  **IL8RB**  which are critical for binding the ligands interleukin 8, NAP 2  [ **PPBP** ]  and GRO alpha.  Ref: 9126332 Biochem Biophys Res Commun, 1997
- This differential phosphorylation by IL 8 and NAP 2  [ **PPBP** ]  of CXCR2  [ **IL8RB** ]  provides a basis for the divergent outcome of PMN induced inflammation in response to these two closely related C X C chemokines.  Ref: 9190946 J Immunol, 1997
- To elucidate the role of CXCR1 and CXCR2  [ **IL8RB** ]  in inflammation and to delineate the basis for the divergent regulation of these receptors by IL 8 and NAP 2  [ **PPBP** ] , we characterized the IL 8 and NAP 2  [ **PPBP** ]  induced mechanisms regulating the expression of each receptor, focusing on receptor internalization and recycling.  Ref: 10623425 Cytokine, 1999
- This was evident from experiments in which inhibition of the CTAP III processing protease and simultaneous blockade of the CXCL7  [ **PPBP** ]  high affinity receptor CXCR 2  [ **IL8RB** ]  led to complete abrogation of CTAP III mediated neutrophil adhesion.  Ref: 12193731 J Immunol, 2002
- A functional hierarchy was shown to exist between GCP 2 and 2 other ELR CXC chemokines, IL 8 and NAP 2  [ **PPBP** ] , in their abilities to induce CXCR1 and CXCR2  [ **IL8RB** ]  internalization, according to the following IL 8 > GCP 2 > NAP 2  [ **PPBP** ] .  Ref: 10688807 Blood, 2000
